# Variants of *WNT7A* and *GPR124* are associated with hemorrhagic transformation following intravenous thrombolysis in ischemic stroke

**DOI:** 10.1111/cns.13457

**Published:** 2020-09-29

**Authors:** Song Ta, Xianfang Rong, Zhen‐Ni Guo, Hang Jin, Peng Zhang, Fenge Li, Zhihuan Li, Lilong Lin, Chenqing Zheng, Qingquan Gu, Yuan Zhang, Wenlan Liu, Yi Yang, Junlei Chang

**Affiliations:** ^1^ Department of Neurology The First Hospital of Jilin University Changchun China; ^2^ Shenzhen Key Laboratory of Biomimetic Materials and Cellular Immunomodulation, Institute of Biomedicine and Biotechnology, Shenzhen Institutes of Advanced Technology Chinese Academy of Sciences Shenzhen China; ^3^ Dongguan Enlife Stem Cell Biotechnology Institute Dongguan China; ^4^ Shenzhen RealOmics Biotech Co., Ltd. Shenzhen China; ^5^ The Central Laboratory, Shenzhen Second People's Hospital, Shenzhen University 1st Affiliated Hospital Shenzhen University School of Medicine Shenzhen China

**Keywords:** blood‐brain barrier, intracerebral hemorrhage, ischemic stroke, signaling pathway, single‐nucleotide polymorphism

## Abstract

**Aims:**

The canonical Wnt signaling pathway plays an essential role in blood‐brain barrier integrity and intracerebral hemorrhage in preclinical stroke models. Here, we sought to explore the association between canonical Wnt signaling and hemorrhagic transformation (HT) following intravenous thrombolysis (IVT) in acute ischemic stroke (AIS) patients as well as to determine the underlying cellular mechanisms.

**Methods:**

355 consecutive AIS patients receiving IVT were included. Blood samples were collected on admission, and HT was detected at 24 hours after IVT. 117 single‐nucleotide polymorphisms (SNPs) of 28 Wnt signaling genes and exon sequences of 4 core cerebrovascular Wnt signaling components (*GPR124*, *RECK*, *FZD4,* and *CTNNB1*) were determined using a customized sequencing chip. The impact of identified genetic variants was further studied in HEK 293T cells using cellular and biochemical assays.

**Results:**

During the study period, 80 patients experienced HT with 27 parenchymal hematoma (PH). Compared to the non‐PH patients, *WNT7A* SNPs (rs2163910, *P* = .001, OR 2.727; rs1124480, *P* = .002, OR 2.404) and *GPR124* SNPs (rs61738775, *P* = .012, OR 4.883; rs146016051, *P* < .001, OR 7.607; rs75336000, *P* = .044, OR 2.503) were selectively enriched in the PH patients. Interestingly, a missense variant of *GPR124* (rs75336000, c.3587G>A) identified in the PH patients resulted in a single amino acid alteration (p.Cys1196Tyr) in the intracellular domain of GPR124. This variant substantially reduced the activity of WNT7B‐induced canonical Wnt signaling by decreasing the ability of GPR124 to recruit cytoplasmic DVL1 to the cellular membrane.

**Conclusion:**

Variants of *WNT7A* and *GPR124* are associated with increased risk of PH in patients with AIS after intravenous thrombolysis, likely through regulating the activity of canonical Wnt signaling.

## 1 INTRODUCTION

Approximately 80% of strokes are caused by thrombotic occlusion of brain arteries with subsequent cerebral ischemia.^1^, ^2^ The only pharmacological intervention approved by the US Food and Drug Administration (FDA) for acute ischemic stroke (AIS) is the thrombolytic agent recombinant tissue‐type plasminogen activator (rt‐PA).^3^, ^4^, ^5^, ^6^ However, rt‐PA treatment significantly increases the risk of hemorrhagic transformation (HT).^7^, ^8^, ^9^, ^10^ As a major complication of thrombolytic therapy, HT especially the parenchymal hematoma (PH) is associated with significantly increased stroke morbidity and mortality.^7^, ^11^ Currently, the risk factors underlying HT pathogenesis remain unclear and clinically amenable biomarkers are urgently needed to better predict the risk and severity of HT.

Brain endothelial cells (BECs) forming the barrier between brain tissue and peripheral blood circulation are called as blood‐brain barrier (BBB) that regulates the molecule diffusion between the blood vessels and brain parenchyma.^12^, ^13^ BECs are the main component of BBB but pericytes, astrocytes, and extracellular basement matrix are also required to BBB function and integrity, which collectively are called neurovascular unit (NVU).^12^, ^14^, ^15^ BECs possess firm intercellular tight junctions (TJs) and a low level of transcytosis, which limit both paracellular and transcellular pathways to transport most substances from blood to the brain.^16^ BBB disruption has been shown to occur in the early reperfusion phase following intravenous thrombolysis and is closely associated with subsequent HT.^17^, ^18^, ^19^, ^20^ Animal and human studies indicate that BBB injury occurs within 1‐2 hours after onset of reperfusion and becomes more severe at 12‐24 hours post‐reperfusion, preceding the appearance of HT.^20^, ^21^, ^22^ Re‐opening of the occluded arteries by rt‐PA or rt‐PA combined with endovascular therapy induces reperfusion injury to microvessels through production of reactive oxygen species (ROS), matrix metalloproteinases (MMPs), as well as other factors from blood and brain cells, which together disrupt the neurovascular unit and cause BBB injury.^7^, ^23^, ^24^, ^25^, ^26^ In addition, rt‐PA may act directly upon endothelial cells, astrocytes, or neutrophils, leading to the activation of PDGF‐CC and MMP‐2/3/9, and thus disrupting BBB function.^18^, ^27^, ^28^, ^29^

We and others have found that canonical Wnt signaling (Wnt/β‐catenin signaling) pathway in brain endothelium is essential to BBB formation and integrity during embryonic development and adulthood in animals. Genetic ablations of canonical Wnt signaling components in mice including the Wnt ligand *Wnt7a/7b* and agonist *Norrin*,^30^, ^31^, ^32^, ^33^ receptor/co‐receptors *Fzd4*, *Lrp5/6*, *Gpr124*, or *Reck*,^31^, ^41^ or effector *Ctnnb1* (β‐catenin),^30^, ^33^, ^39^, ^42^, ^43^ compromises BBB formation or integrity, accompanied by widespread or focal intracerebral hemorrhage (ICH). Recently, we demonstrated that following acute cerebral ischemia/reperfusion injury in mice, decreased canonical Wnt signaling activity in *Gpr124* knockout brain endothelium led to exacerbation of BBB disruption and severe ICH, resembling severe PH in human strokes.^21^ Furthermore, canonical Wnt signaling activity regulated rt‐PA‐induced HT in rodent ischemic stroke models.^44^, ^45^, ^46^ Nevertheless, the impact of canonical Wnt signaling pathway on human BBB integrity and HT after ischemic stroke remains completely unexplored. In the current study, we aimed to identify the association of the canonical Wnt signaling with risk of HT in AIS patients by assessing sequence variations in Wnt signaling genes and determine their effects on the activity of cellular Wnt signaling.

## 2 METHODS

The de‐identified participant data generated and analyzed in the current study will be available and shared at request of other investigators for purposes of replicating procedures and results.

### 2.1 Standard protocol approvals and patient consents

Written informed consent was obtained from each patient included in the study prior to enrollment. The study protocol conforms to the ethical guidelines of the 1975 Declaration of Helsinki and has been approved by the Ethics Committee of the First Hospital of Jilin University prior to initiation of the study.

### 2.2 Participants

From June 2015 through May 2018, consecutive AIS patients in the First Hospital of Jilin University receiving intravenous thrombolysis were screened for eligibility and enrolled. Enrollment criteria were as follows: (a) Blood samples were collected at admission prior to rt‐PA administration; (b) CT scans performed both at admission and 24 hours after thrombolysis. Under these selection criteria, 355 thrombolysis patients were included in this study. Due to the lack of previous studies identifying the frequencies of the Wnt signaling genetic variants in ischemic stroke patients, we were not able to perform power analysis to estimate the approximate number of patients needed before the study began. Therefore, we performed an interim analysis using the data of the first group of patients recruited during the study and estimated the total patient number required (Tables S1 and S2). Furthermore, a power analysis was performed after study completion.

### 2.3 Thrombolytic procedure

Acute ischemic stroke patients were considered for the use of rt‐PA thrombolytic therapy, with the following conditions: (a) age > 18 years; (b) presentation within 4.5 hr of symptom onset; (c) ruled out for intracranial and subarachnoid hemorrhage. Clinical examination, blood and coagulation tests, 12‐lead electrocardiogram (ECG), fingertip blood sugar, and blood biochemistry test were performed at admission prior to rt‐PA administration. Rt‐PA was administered according to the relevant institutional protocols and international recommendations (0.9 mg/kg of estimated or measured body weight, 10% as a bolus and 90% as an infusion over a period of 60 minutes, and a maximal dose of 90 mg). Stroke severity was assessed using National Institute of Health Stroke Scale (NIHSS) score by certified vascular neurologists at admission and at 1, 3, and 7 days post‐rt‐PA administration. We recorded relevant medical history including hypertension, atrial fibrillation, and history of taking antiplatelet drugs. All patients were admitted to an acute stroke unit and monitored continuously.

### 2.4 Hemorrhagic transformation detection

All thrombolytic patients received CT scans at 24 hr after thrombolysis. The CT scan results were evaluated by a professional neurologist blinded to the clinical characteristics of the stroke and its progress. Hemorrhagic transformation was classified according to the European Cooperative Acute Stroke Study I (ECASS I),^11^ including (a) no hemorrhagic transformation; (b) hemorrhagic infarction 1 (HI1): scattered small petechiae, no mass effect; (c) hemorrhagic infarction 2 (HI2): more confluent petechiae, no mass effect; (d) parenchymal hematoma 1 (PH1): hematoma within infarcted tissue, occupying <30% of the infarcted area, mild mass effect; and (e) parenchymal hematoma 2 (PH2): hematoma occupying >30% of the infarcted tissue, with significant mass effect.

### 2.5 Blood collection and DNA extraction

Venous blood samples were collected at admission from patients prior to rt‐PA administration as a standard of care. After collection, samples were centrifuged at 4°C, 1811 *g*, for 10 minutes. Serum and leukocytes were immediately frozen and stored at −80°C until further the analyses were performed. Genomic DNA of isolated leukocytes was extracted with the GOMag Blood DNA Kit from GeneOn BioTech (Germany, Cat. #GO‐HYAS) following the manufacturer's instructions.

### 2.6 Detection of single‐nucleotide polymorphisms and gene sequence variations

**Figure 1:**
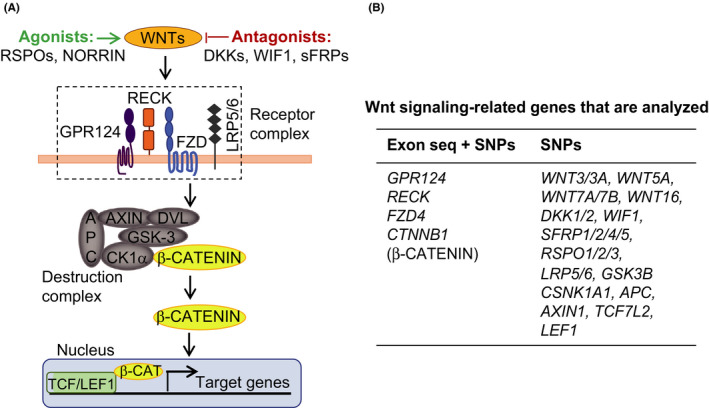
Schematic diagram of the study design. A, Schematic illustration of the canonical Wnt signaling in brain endothelium. B, Summary of Wnt signaling‐related genes assessed in this study. Either both exon sequence variations and known SNPs (left column) or only known SNPs (right column) were determined and analyzed

In total, 117 single‐nucleotide polymorphisms (SNPs) and genetic variants of 28 Wnt signaling pathway‐related genes were reported by previous studies and thus summarized after searching the genome‐wide association studies (GWAS) Catalog of the European Bioinformatics Institute (https://www.ebi.ac.uk/gwas/) (Figure 1). As the sequencing probes were ~100 bp long, additional SNPs that were proximal to the identified SNPs in location in the DNA sequences were also detected. Full‐length exon sequencing for *GPR124*, *RECK*, *FZD4*, and *CTNNB1* (gene for β‐CATENIN) was performed additionally to identify any potential sequence variations or mutations not reported previously. Please see Appendix S1 for detailed protocols.

### 2.7 Plasmid construction, Western blotting, immunofluorescence, immunoprecipitation, and TOP‐Flash assays

Wild‐type (WT) *GPR124*, *GPR124* with a single‐nucleotide mutation (c.3587G>A, ie, p.Cys1196Tyr), and *GPR124* with an intercellular domain deletion (GPR124 △ICD) were cloned into the pcDNA3.1 vector with an N‐terminal 3xFLAG. DVL1/2/3, WNT7B, and FZD4 were cloned into the pcDNA3.1 vector with C‐terminal tags (6xHis tag, HA tag, and Myc tag, respectively). Plasmids were overexpressed in HEK 293T cells. Protein expression was determined by Western blotting, and subcellular location was determined by confocal immunofluorescence staining. Protein interaction between GPR124 and DVL was determined by co‐immunoprecipitation (co‐IP) or co‐immunofluorescence staining (co‐IF). Wnt signaling activity was determined by *Axin2* qRT‐PCR or β‐CATENIN‐dependent luciferase transcription assay (TOP‐Flash). Please see Appendix S1 for detailed protocols.

### 2.8 Statistical analysis

Patient data were analyzed using the Statistical Package for the Social Sciences, version 22.0 (SPSS, IBM Corporation). PLINK1.9 was performed to check principal components analysis (PCA) based on the variance‐standardized relationship matrix for population stratification and Hardy‐Weinberg equilibrium deviation. Single mark association analysis for qualified high‐confidence datasets was performed to compute the odds ratios (ORs) and *P*‐values in PLINK using Fisher's exact test for dichotomous phenotypes. The continuous variables were presented as mean ± SD or median (interquartile range), depending on the distribution of the variable. The Shapiro‐Wilk test was used to test the normality of the data. The Mann‐Whitney test or Student's *t* test was used to compare the differences between two independent samples. Comparison of the differences between three independent samples was conducted by Kruskal‐Wallis test. Pairwise comparisons of groups were corrected by the way of Bonferroni correction. Interim analysis to estimate the total patient number and power analysis were done with PASS 11.0. The count data were expressed as the rate (percentage) and analyzed with the chi‐square and Fisher exact tests. All tests were two‐tailed and were considered statistically significant when *P* < .05 (unless specifically noted).

## 3 RESULTS

### 3.1 Patient characteristics and hemorrhagic transformation

During the study period, we consecutively enrolled 355 eligible AIS patients treated with intravenous rt‐PA. Males comprised 74.1% of patients, and the mean age of the patients was 62.88 ± 11.96 years. Median [quartiles] NIHSS score on admission was 9 [5,13], and mean time to treatment from symptoms onset was 188.86 ± 63.16 min. HT was observed in 80 (22.5%) patients, of which there were 53 (14.9%) patients with HI, 27 (7.6%) patients with PH, and 4 (1.1%) patients with symptomatic HT (all PH).

Because only PH but not HI is associated with early neurological deterioration and increased patient mortality,^11^ we focused on analyzing the PH versus non‐PH patients. A summary of the demographic and baseline characteristics of these patients is described in Table 1, grouped by PH versus non‐PH.

**Table 1:** Demographic and baseline clinical characteristics by parenchymal hematoma (PH) groups

Factors	Non‐PH group (n = 328)	PH group (n = 27)	*χ* ^2^ (*t*, *z*)	*P*
Male	241 (73.5%)	21 (77.8%)	0.239	.661
Age (y)	62.77 ± 12.01	64.00 ± 10.97	−0.512	.609
BMI	24.22 (23.23‐24.69)	23.87 (22.76‐24.49)	−1.518	.129
Hypertension	172 (52.4%)	15 (55.6%)	0.097	.842
Coronary artery disease	103 (31.4%)	5 (18.5%)	1.956	.195
Atrial fibrillation	34 (10.4%)	4 (14.8%)	0.517	.512
Antiplatelet therapy	21 (6.4%)	4 (14.8%)		.110
History of stroke	68 (20.7%)	4 (14.8%)	0.540	.621
Baseline NIHSS	9 (5‐12)	10 (5‐15)	−1.178	.239
Baseline mRS	4 (3‐4)	4 (3‐4)	−0.672	.502
OTT (onset to treatment, min)	187.13 ± 59.83	199.48 ± 62.49	−1.028	.305
Platelet count (10^9^/L)	194.50 (165.00‐229.75)	201.00 (153.25‐238.25)	−0.177	.859
INR	0.94 (0.90‐0.99)	0.94 (0.91‐0.97)	−0.630	.529
Baseline left systolic blood pressure (mmHg)	150.80 ± 21.11	154.56 ± 14.79	−0.905	.609
Baseline left diastolic blood pressure (mm Hg)	89.00 (80.00‐97.00)	88.00 (80.00‐97.00)	−0.330	.742
Baseline blood glucose (mmol/L)	7.7 (6.5‐9.4)	8.9 (6.9‐10.9)	−1.952	.051
Hyperlipidemia	26 (7.9%)	2 (7.4%)		1.000
Total cholesterol (mmol/L)	4.82 (4.19‐5.53)	4.52 (3.63‐5.76)	−1.262	.207
LDL (mmol/L)	2.88 (2.45‐3.48)	3.20 (2.11‐3.51)	−0.042	.966
Triglyceride (mmol/L)	1.35 (0.93‐2.14)	1.36 (1.05‐1.77)	−0.474	.635

Note

Data were presented as mean ± SD or median (interquartile range).

Abbreviations: BMI, body mass index; HT, hemorrhagic transformation; INR, international normalized ratio; LDL, low‐density lipoprotein; NIHSS, National Institutes of Health Stroke Scale.

### 3.2 Associations of genetic variations with HT

We thoroughly searched the GWAS Catalog of the European Bioinformatics Institute and identified 117 SNPs after merging the overlapping SNPs for 28 Wnt signaling‐related genes (Figure 1, and full SNP list is available upon request). Additionally, we performed full‐length exon sequencing for *GPR124*, *RECK*, *FZD4,* and *CTNNB1* (gene for β‐CATENIN), which represent four core components of the Wnt signaling in the cerebrovasculature, to identify any potential sequence variations or mutations previously unknown to be linked with Wnt signaling‐related phenotypic traits.

Our results showed that compared to the non‐PH patients, five genetic variants belonging to *Wnt7A* and *GPR124* were selectively enriched in the PH patients, including two SNPs of *WNT7A* (rs2163910, *P* = .001, odds ratio [OR] 2.727; rs1124480, *P* = .002, OR 2.404) and three SNPs of *GPR124* (rs61738775, *P* = .012, OR 4.883; rs146016051, *P* < .001, OR 7.607; rs75336000, *P* = .044, OR 2.503) (Table 2). Two SNPs of *RECK* were also found to be enriched in the PH patients but did not reach statistical significance (rs12235235, *P* = .090, OR 1.882; rs2274522, *P* = .089, OR 1.936), likely due in part to the limited number of PH patients included in this study, as shown by the interim analysis result during the study (Tables S1 and S2) and power analysis after the study with power values below 0.8 (Table 2). Consistent with the overall benign nature of HI in clinic, we did not identify any genetic variants that were significantly enriched in the HI patients as compared to the non‐HT patients (data not shown).

**Table 2:** Association of Wnt signaling genetic variants with HT by PH groups

Gene name	dbSNP ID	Base change	Variant type or location	Allele Frequency		OR	*P*‐value	Power
				Non‐PH (%)	PH (%)			
*WNT7A*	rs2163910	C>A	3′‐UTR variant	36.8	61.4	2.727	.0012	0.9463
	rs1124480	T>C	3′‐UTR variant	23.4	42.3	2.404	.0024	0.8409
*GPR124*	rs61738775	G>A	Synonymous variant	1.2	5.8	4.883	.0116	0.6488
	rs146016051	G>A	Intron variant	1.1	7.7	7.607	.0002	0.8062
	rs75336000	G>A	Missense variant (c.3587G>A)	4.9	11.5	2.503	.0441	0.5313
*RECK*	rs12235235	G>A	Intron variant	13.5	22.7	1.882	.0903	0.4676
	rs2274522	G>A	Intron variant	11.7	20.5	1.936	.0887	0.4770

Abbreviations: dbSNP, database single‐nucleotide polymorphism; HT, hemorrhagic transformation; OR, odds ratio; PH, parenchymal hematoma; UTR, untranslated region.

**Figure 2:**
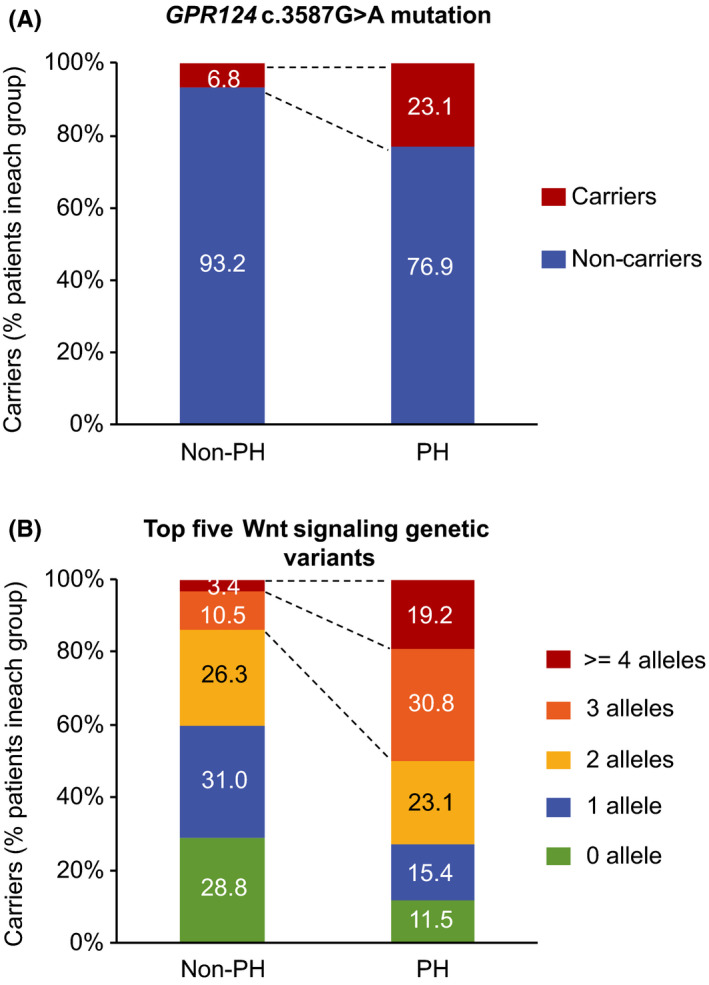
Shift distribution of patient numbers by *GPR124* c.3587G>A mutation or by the top five Wnt signaling genetic variants. Number of patients (%) in each group with *GPR124* c.3587G>A mutation (A) or with alleles of the top five Wnt signaling genetic variants enriched in the PH patients (*WNT7A* rs2163910 and rs1124480, *GPR124* rs61738775, rs146016051, and rs75336000; 2 alleles/variant, 10 alleles totally) (B) was calculated

Most of these variants were located in introns or downstream of the gene coding regions, with the exception of a missense variant of *GPR124* (rs75336000, c.3587G>A) that is located in exon 19 and yielding an amino acid change (p.Cys1196Tyr) of GPR124 protein. Further analysis showed that 23.1% PH patients carried *GPR124* c.3587G>A mutation compared to only 6.8% carriers in non‐PH patients (Figure 2A), suggesting a potential role of this point mutation in the pathogenesis of HT in at least a subset of AIS patients.

In animal genetic studies, Wnt signaling components have been found to act cooperatively or redundantly in the regulation of BBB integrity and ICH.^32^, ^39^ Therefore, we analyzed the top five genetic variants of *Wnt7A* and *GPR124* genes listed above collectively in order to evaluate their accumulative effects on risk of HT. Compared to the non‐PH group, a significantly higher percentage of PH patients carried either both of the *WNT7A* variants rs2163910/rs1124480 (63.6% in PH versus 30.7% in non‐PH), or 3 alleles (30.8% in PH versus 10.5% in non‐PH) or >=4 alleles (19.2% in PH versus 3.4% in non‐PH) of the top five Wnt signaling genetic variants (2 alleles/variant, 10 alleles in total; Figure 2B). These data suggest an incremental effect of copy number of these variants on the risk of severe HT.

### 3.3 *GPR124* c.3587G>A mutation reduces Wnt signaling activity by promoting dissociation of cytosolic DVL1 from GPR124

**Figure 3:**
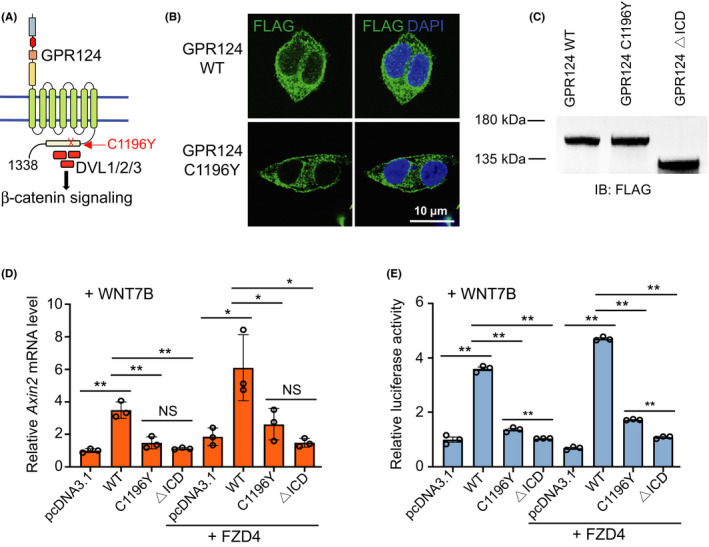
The GPR124 C1196Y mutation reduces Wnt signaling activity. A, Schematic illustration of the C1196Y alteration in the intracellular domain (ICD) of GPR124. B, Wild‐type (WT) GPR124 and GPR124 C1196Y were cloned into pcDNA3.1‐3xFLAG construct and overexpressed in HEK 293T cells. Protein subcellular location was determined by confocal immunofluorescence staining. C, Expression levels of GPR124 WT, GPR124 C1196Y, and GPR124 with ICD deletion (GPR124 △ICD) were determined by Western blotting. D‐E, Wnt signaling activity was measured by *AXIN2* qRT‐PCR (D) and β‐catenin‐dependent transcription assay TOP‐Flash (E) after transfecting HEK 293T cells with the indicated plasmids. Data are presented as mean ± SE. *P < .05, **P < .01. NS, not significant, Student's *t* test

We next examined how the single‐nucleotide mutation (c.3587G>A) of *GPR124* affects its protein function and Wnt signaling activity. This nucleotide mutation causes a single amino acid change in the intercellular domain (ICD) of GPR124, that is, p.Cys1196Tyr or C1196Y (Figure 3A). Therefore, we cloned *GPR124* wild‐type (WT) and *GPR124* C1196Y into pcDNA3.1‐3xFLAG plasmid and overexpressed them in HEK 293T cells. The C1196Y mutation did not change the subcellular location or expression level of GPR124 (Figure 3B‐C). However, this mutation substantially reduced Wnt signaling activity to levels nearly similar to those of empty plasmid control or GPR124 ΔICD, as measured by Wnt target gene *Axin2* mRNA levels and β‐catenin‐dependent transcription assay (Figure 3D‐E).

**Figure 4:**
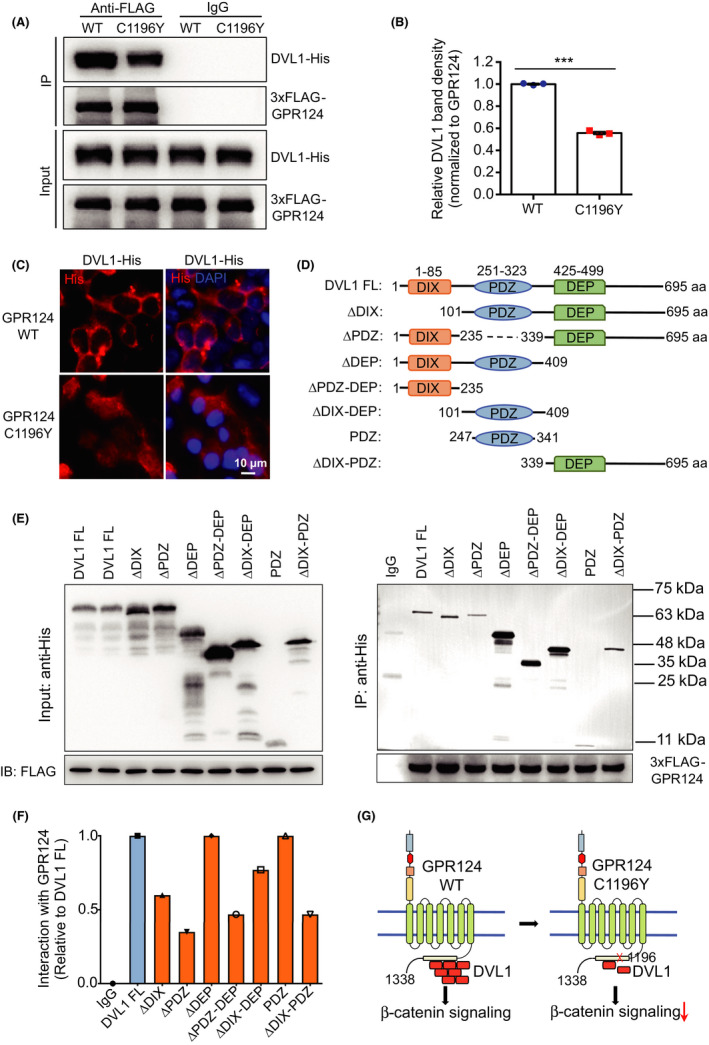
The GPR124 C1196Y mutation decreases the interaction between DVL1 and GPR124. A, 3xFLAG‐GPR124 WT or 3xFLAG‐GPR124 C1196Y was co‐expressed with DVL1‐His in HEK 293T cells. GPR124 was precipitated with anti‐FLAG antibody, and DVL1 protein bound to GPR124 was detected with anti‐6xHis antibody. This experiment was repeated three times and quantified in (B). Data are presented as mean ± SE. ***P < .01, Student's *t* test. C, Interaction between GPR124 (WT or C1196Y) and DVL1 was determined by co‐immunofluorescence staining (co‐IF) in HEK 293T cells. D‐F, A series of truncated DVL1 mutants were constructed, and co‐IP was performed to map the binding sites of DVL1 with GPR124 protein. Experiments were repeated three times, and representative results were presented. G, Proposed working model

The ICD of GPR124 has been shown to potentiate WNT7 signaling by recruiting cytosolic DVL protein to the cellular membrane and thus increase the interaction between DVL and Wnt receptors FZDs and LRP5/6.^47^ Our data showed that GPR124 interacted with DVL1 but not DVL2 or DVL3 in human HEK 293T cells (Figure 4A‐B and Figure S1). Furthermore, the C1196Y mutation of GPR124 significantly decreased the interaction between GPR124 and DVL1, and largely abolished the membrane recruitment of cytosolic DVL1 by GPR124 ICD (Figure 4A‐C). Lastly, we constructed a series of truncated DVL1 mutants and performed co‐IP assays to map the binding sites of DVL1 with GPR124 (Figure 4D). Our data showed that the interaction between DVL1 and GPR124 involved all the three domains (DIX, PDZ, and DEP) of DVL1, wherein the PDZ domain played a more prominent role than the DIX or DEP domain (Figure 4E‐F).

## 4 DISCUSSION

In this study, we have extensively examined the association of genetic variants of canonical Wnt signaling pathway with the risk of HT following rt‐PA‐mediated thrombolysis therapy in a cohort of Chinese AIS patients. We identified that several genetic variants of *WNT7A* and *GPR124* are associated with risk of parenchymal hematoma (PH), the clinically detrimental form of HT. We further demonstrated the mechanisms of how these genetic variants may affect cellular canonical Wnt signaling activity, focusing on the *GPR124* c.3587G>A mutation. To the best of our knowledge, this is the first report of an association of Wnt signaling pathway variants with human BBB integrity and intracerebral hemorrhage after ischemic stroke, setting an effective example for translating findings of animal studies to human clinical practice.

Previous studies have shown that early HT following a cerebral infarct occurs in as many as 10% to 40% of patients receiving rt‐PA with or without endovascular therapy.^8^, ^48^ Patients with PH present significantly worse neurological outcomes and a higher mortality rate.^8^, ^11^ However, the pathogenesis of HT remains unclear and no reliable indicators can predict HT occurrence, which have been profoundly limiting the application of rt‐PA to AIS patients. BBB disruption is reported numerously by many groups following reperfusion therapy in both animal stroke models and AIS patients and is shown to be a major contributing factor of HT.^7^, ^17^, ^18^, ^19^, ^20^, ^23^, ^49^ The essential role of cerebrovascular Wnt/β‐catenin signaling in BBB formation and integrity has been well documented by us and others in animal studies. Impaired Wnt/β‐catenin signaling in mouse embryos led to severe BBB disruption and intracerebral hemorrhage by altering expressions of BBB markers Glut‐1, Claudin‐5, and Plvap.^30^, ^33^, ^34^, ^39^, ^43^ In adult mice, we recently reported that impaired Wnt/β‐catenin signaling adversely impacted BBB integrity was unveiled during cerebral ischemia/reperfusion injury and glioma angiogenesis.^21^, ^50^ A previous study suggested that disruption of the nuclear localization of endothelial β‐catenin was associated with hemorrhagic lesions in hemorrhagic stroke patients.^42^ However, evidence supporting a role for cerebrovascular Wnt signaling in human BBB function and intracerebral hemorrhage during ischemic stroke has been lacking. Our results demonstrated that *WNT7A* and *GPR124* variants were significantly associated with the risk of HT following thrombolytic reperfusion therapy in AIS patients, suggesting that they may be involved in the pathogenesis of HT in humans. Of note, *GPR124* variants have a low allele frequency; thus, these variants likely contributed to HT in a subset of AIS patients. Overall, our study is the first to reveal a potential role for the cerebrovascular Wnt signaling in BBB disruption and HT in at least a subset of ischemic stroke patients.

WNT7A and WNT7B are two major WNT ligands expressed in mouse brain and act to maintain the Wnt/β‐catenin signaling in brain endothelium.^30^, ^33^ In addition, the adhesion G protein‐coupled receptor GPR124 and the membrane‐anchored glycoprotein RECK function cooperatively to specifically potentiate cerebrovascular WNT7 signaling by presenting WNT7 to the FZD receptor extracellularly and recruiting cytosolic DVL to the cell membrane intracellularly.^47^, ^51^ In mouse embryos, double knockouts (but not single knockouts) of *Wnt7a*/*Wnt7b* exhibit CNS‐wide parenchymal hemorrhage,^30^ while knockout of either *Gpr124* or *Reck* results in forebrain and spinal cord‐restricted hemorrhage.^34^, ^41^ Our sequencing data in AIS patients suggest that WNT7 and particularly GPR124 may also play a role in human BBB function regulation. In this study, two variants of *WNT7A* within the 3′ untranslated region (3′‐UTR variants) were identified to be associated with PH. These two variants may affect the expression levels of *WNT7A* by regulating its mRNA localization, stability, and translation,^52^ which warrant further studies. In our previous study, we have demonstrated a key role of GPR124 in the occurrence of intracerebral hemorrhage following cerebral ischemia/reperfusion injury in mice.^21^ Notably, here we identify a missense variant of *GPR124* gene (c.3587G>A) that results in a single amino acid change (C1196Y) in the ICD of GPR124 protein, and this variation is significantly enriched in PH patients. Using cellular and biochemical assays, we show that the C1196Y mutation significantly decreases the interaction between GPR124 and DVL1, then almost abolishes the membrane recruitment of cytosolic DVL1 by GPR124, and eventually reduces downstream β‐catenin signaling (Figure 4G). Lastly, we found a copy number incremental effect of different Wnt genetic variants on the risk of HT, suggesting that combined analysis of multiple Wnt signaling genetic variants may better predict the risk of severe PH following thrombolysis therapy in AIS patients.

We acknowledge that there are limitations in this study. First, the study was performed in a single hospital and all patients were Chinese, but doing so may help to ensure the consistency of rt‐PA treatment and diagnosis of HT following thrombolysis and reduce the influence of genetic background differences. Second, the sample size was relatively small, which was due to the low number of AIS patients eligible for rt‐PA therapy in clinic and low occurrence rate of PH in AIS patients. Studies with a larger number of AIS patients from multiple hospitals are needed to further validate our findings. Third, the Wnt SNPs examined in this study were identified using genomic DNA from peripheral blood leukocytes; whether WNT7A and GPR124 are also expressed in these cells and affect HT remains to be determined.

In summary, our findings suggest a role of the canonical Wnt signaling pathway in rt‐PA‐mediated BBB disruption and HT in a subset of AIS patients. The biomarkers reported here may be used in conjunction with other biomarkers reported previously to assist in identifying AIS patients at higher risk for HT and guide the thrombolysis treatment and care of these patients.

## CONFLICT OF INTEREST

The authors declare no conflict of interest.

## Supporting information

Appendix S1

## Data Availability

The datasets generated and analyzed during the current study are available from the corresponding authors on reasonable request.
