# Supplementary material for: Variants of WNT7A and GPR124 are associated with hemorrhagic transformation following intravenous thrombolysis in ischemic stroke
Source: CNS Neurosci Ther. 2020 Sep 29;27(1):71–81. doi: 10.1111/cns.13457 (PMC7804912; doi:10.1111/cns.13457)

SUPLLEMENTAL MATERIAL

Variants of *WNT7A* and *GPR124* are associated with hemorrhagic transformation following intravenous thrombolysis in ischemic stroke

Song Ta[1,2] *, Xianfang Rong[2] *, Zhen-Ni Guo[1], Hang Jin[1], Peng Zhang[1], Fenge Li[1], Zhihuan Li[3], Lilong Lin[3], Chenqing Zheng[4], Qingquan Gu[4], Yuan Zhang[5], Wenlan Liu[5], Yi Yang[1 #], Junlei Chang[2 #]

Supplemental Methods

Detection of Gene Sequence Variations and Polymorphism

Target sequencing

Qualified genomic DNA for each individual was hybridized with the designed target capture array including customized probes to enrich target DNA in each library. We performed sequencing on the Illumina X Ten platform with 150 bp pair reads independently for each captured library to ensure that each sample had average coverage of ~700-2600 fold.

Data Pre-processing

Samples were aligned to the NCBI human genome reference assembly (hg19) using BWA (Burrows-Wheeler Aligner). Next, we perform Picard Mark Duplicates to mark the duplicates reads to mitigate biases introduced by data generation such as PCR amplification. The BAM files were processed, using the Genome Analysis Toolkit (GATK v3.3) to perform realignment around known indels, and we then recalibrate the base quality scores for the individual base calls in each sequence read.

Variant Discovery

Germline short variant discovery proceeds from analysis-ready BAM files and produces variant calls. GATK(v3.3) HaplotypeCaller was used to call variants per sample in targeted and flanking regions for each individual in order to produce a file in GVCF format. We then perform joint genotyping, to combine the multisample GVCF. Next, we perform GenotypeGVCFs to get multisample genotype for all sites, and finally, hard-filter was applied to produce the final multisample callset with the desired balance of precision and sensitivity.

Annotation

SnpEff was used to separate single-nucleotide variations (SNVs) into different functional categories according to their genic location and their expected effect on encoded gene products, based on information from the RefSeq database. All variants were further annotated by the control population of the 1000 Genomes Project (2014 Oct release, http://www.1000genomes.org), ExAC (http://exac.broadinstitute.org), EVS (http://evs.gs.washington.edu/EVS), Disease databases of ClinVar (http://www.ncbi.nlm.nih.gov/clinvar), OMIM (http://www.omim.org). In addition, we categorized the SNVs into known or novel according to whether they were present in dbSNP (version 150).

Data Analysis

We used GATK CombineGVCFs to combine target sequencing dataset with ethnically matched and unrelated subjects in HT cases (HI and PH) and non-HT controls. Then,

the qualified datasets were employed for statistical analysis. PLINK1.9 was performed to check the dataset: principal components analysis (--PCA) extracts the top 10 principal components of the variance-standardized relationship matrix for Population stratification and Hardy-Weinberg equilibrium exact deviation was calculated(--hardy). After filtered unqualified variants (--biallelic-only --geno 0.2 --hwe 0.0001), single-variant association analysis for SNVs and inDel were performed to compute the the odds ratios(ORs) and *P* values by case-control association analysis with fisher model using PLINK 1.9.

Plasmid construction

Human GPR124 WT (N-terminal 3xFLAG tag), Human GPR124 C1196Y (N-terminal 3xFLAG tag) and a series of truncated Human DVL1 (C-terminal 6xHis tag) were synthetized and cloned into the pcDNA3.1 expression vector by Jinweizhi (Suzhou, China). GPR124 △ICD (△aa1078-1338, N-terminal 3xFLAG tag), Human FZD4 (C-terminal Myc tag), WNT7B (C-terminal HA tag) and Human DVL1, DVL2 and DVL3 (C-terminal 6xHis tag) were cloned into pcDNA3.1 expression.

Western blotting

Western blotting was performed using standard method. Briefly, samples (or eluted from magnetic beads) were run on polyacrylamide gels, transferred to PVDF membranes (EMD Millipore, Hayward, CA), blocked with 5% BSA (Sigma-Aldrich), and incubated with the indicated primary antibodies (1:1000) overnight at 4℃. After washed with TBST, the membranes were incubated with secondary antibodies (1:10000) at room temperature for 2 hours. The signals were detected using a chemiluminescence (ECL) kit, scanned using GelView 6000M system and analyzed by densitometric evaluation using the Image J.

Immunofluorescence

HEK 293T cells were seeded into PDL-coated 8-chamber glass slides and incubated for 24 hrs. Cells were transfected with 5 ng of various expression plasmids for 48 hrs and fixed with 4% formaldehyde in PBS for 20 min. Fixed cells were washed 3 times with PBS, incubated with 0.2% Triton X-100 for 10 min, blocked with 5% normal goat serum in PBS for 1 hr and incubated with anti-3xFLAG or anti-His antibody in 1% normal goat serum overnight at 4℃. Cells were washed as above and incubated with Alexa Fluor 488-conjugated goat anti-mouse or Alexa Fluor 594-conjugated goat anti-rabbit antibody (Jackson ImmunoResearch, 1:500) in 1% normal goat serum for 1 hr. Cells were washed as before and covered with anti-fade medium with DAPI, followed by imaging using a fluorescence microscope (CKX53, Olympus, Germany).

Immunoprecipitation

For co-immunoprecipitation assays, HEK 293T were collected 48 hrs after transfection by using RIPA buffer. The supernatant was incubated with anti-3xFLAG antibody (Sigma) overnight at 4℃. Protein G Magnetic beads (Cell Signaling Technology) were incubated with the supernatant for 2 hrs at 4℃, followed by washing five times with the lysis buffer and boiled in 2x Laemmli Sample buffer.

Quantitative PCR (qPCR)
Total RNA was extracted using Direct-zol™RNA MiniPrep (Zymo research), and reverse transcribed using Hiscript II Q RT SuperMix for qPCR (Vazyme). The qPCR was performed by using ChamQ™ Universal SYBR qPCR Master Mix(Vazyme) for amplifying certain PCR products. Expressions of the target genes were analyzed by relative expression ratio between the genes and GAPDH. All reactions were performed in triplicate. The primers used are shown as follows:
Human *AXIN2* Forward: CAACACCAGGCGGAACGAA;
Human *AXIN2* Reverse: GCCCAATAAGGAGTGTAAGGACT;
Human *GAPDH* Forward: TGTGGGCATCAATGGATTTGG;
Human *GAPDH* Reverse: ACACCATGTATTCCGGGTCAAT.

TOP-Flash assay
The Firefly/Renilla dual luciferase reporter assay (Promega) was used to determine the transcriptional activity of Wnt/β-catenin signaling pathway. HEK 293T cells stably expressing the reporter plasmids (firefly luciferase and renilla luciferase) were seeded in a 96-well plate and transient transfection was performed with Fugene HD (Promega) for various GPR124 and DVL1/2/3 expressing plasmids. After 48 hrs, cells were collected and luciferase activity was measured using the Promega Dual-Luciferase Reporter Assay System. The β-catenin responsive firefly luciferase activity of each sample was normalized to Renilla luciferase activity and presented as ratio to empty plasmid controls.

Supplemental Tables

Supplemental Table 1. Interim analysis of the association of Wnt signaling genetic variants with HT by PH groups (96 non-PH, 10 PH).

Gene name	dbSNP ID	Base Change	Variant Type or location	Allele Frequency		OR	*P* value
				Non-PH (%)	PH (%)		
WNT7A	rs2163910	A>C	3'-UTR variant	36.9	60.0	2.568	0.0460
	rs1124480	T>C	3'-UTR variant	24.5	55.5	3.771	0.0036
GPR124	rs61738775	G>A	Synonymous variant	1.0	10.0	10.56	0.0051
	rs146016051	G>A	Intron variant	1.0	10.0	10.56	0.0051
	rs75336000	G>A	Missense variant (p.Cys1196Tyr)	2.6	20.0	9.35	0.0002
RECK	rs12235235	G>A	Intron variant	10.0	20.0	2.25	0.1797
	rs2274522	G>A	Intron variant	8.1	20.0	2.827	0.0869

Abbreviations: dbSNP = database single-nucleotide polymorphism; HT = hemorrhagic transformation; OR = odds ratio; PH = parenchymal hematoma; UTR, untranslated region.

Supplemental Table 2. Total patient number estimation based on the data in Supplemental Table 1. Set the Power as 80%, α as 0.05.

Total patient number	PH number	Non-PH number	dbSNP ID	Allele Frequency in PH (10)	Allele Frequency Non-PH (96)
212	20	192	rs2163910	60.0%	36.9%
106	10	96	rs1124480	55.5%	24.5%
191	18	173	rs61738775	10.0%	1.0%
191	18	173	rs146016051	10.0%	1.0%
106	10	96	rs75336000	20.0%	2.6%
520	49	471	rs12235235	20.0%	10.0%
340	32	308	rs2274522	20.0%	8.1%

Supplementary Figure 1.

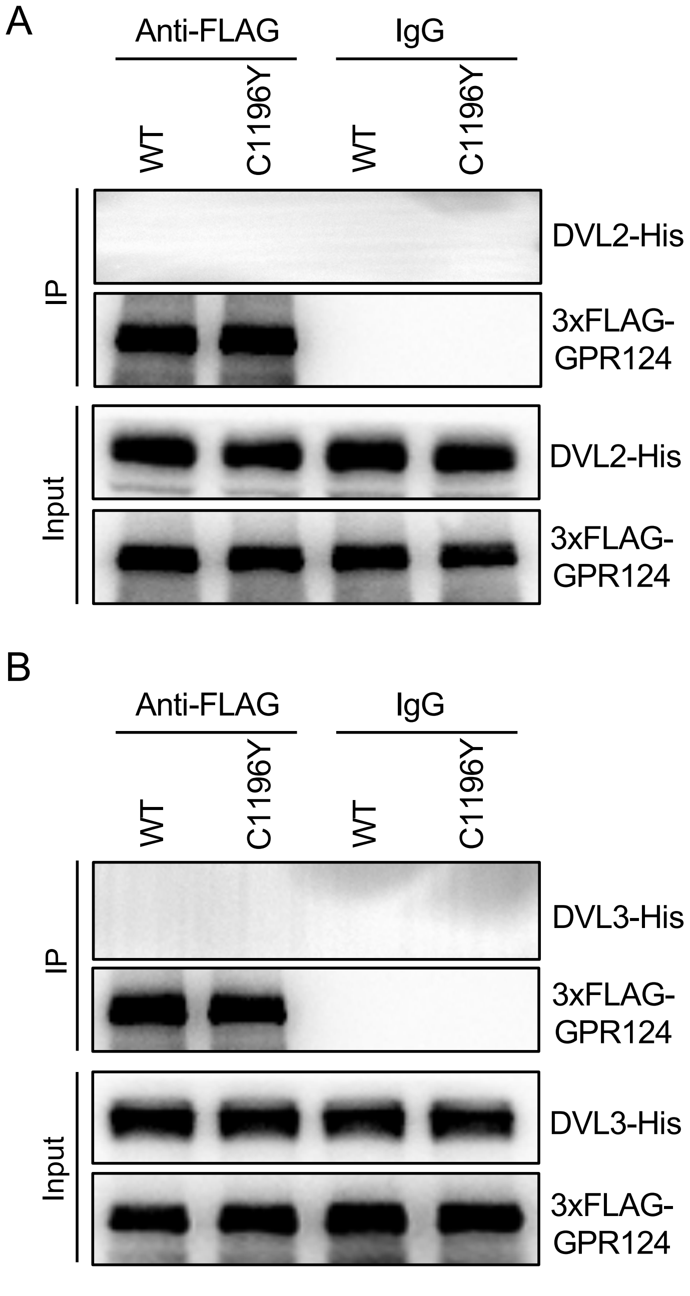

Supplementary figure 1. Effect of GPR124 C1196Y mutation on the interaction between DVL2/3 and GPR124. 3xFLAG-GPR124 WT or 3xFLAG-GPR124 C1196Y were co-expressed with DVL2-His (**A**) or DVL3-His (**B**) in HEK 293T cells. GPR124 was precipitated with anti-FLAG antibody and DVL2 or DVL3 protein bound to GPR124 was detected with anti-6xHis antibody.

Full uncropped gel/blot for Figure 3C

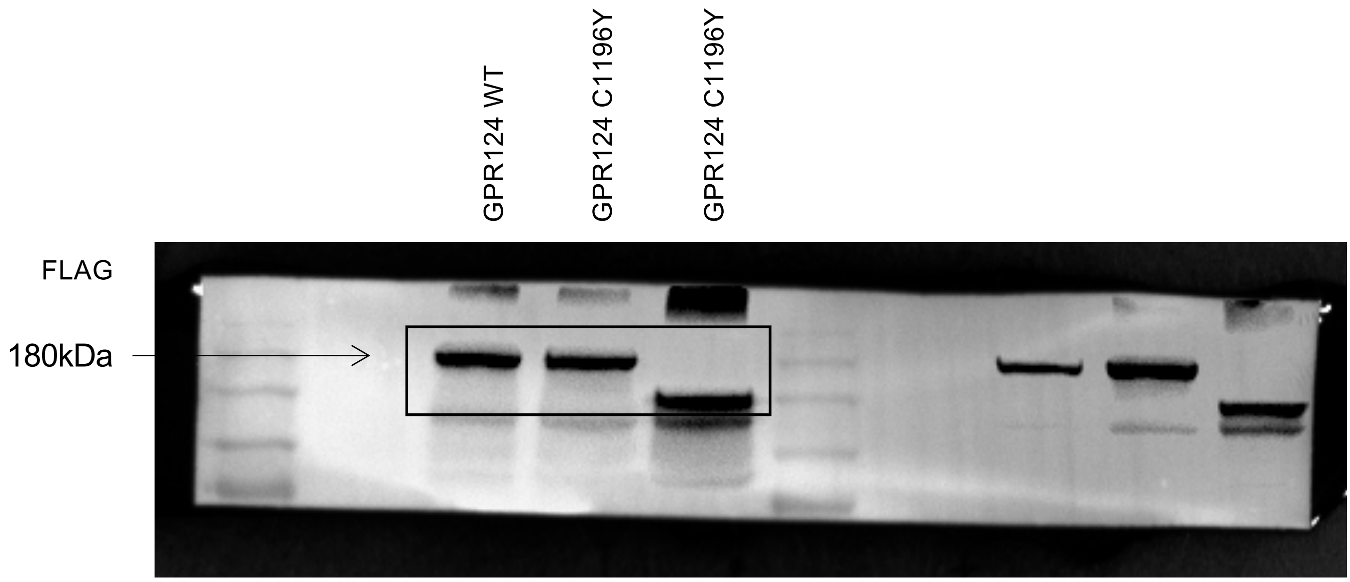

Full uncropped gel/blot for Figure 4A

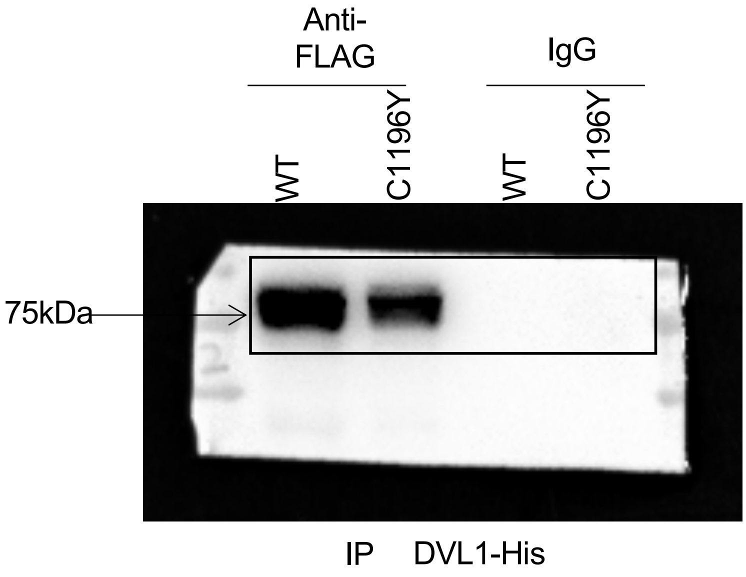

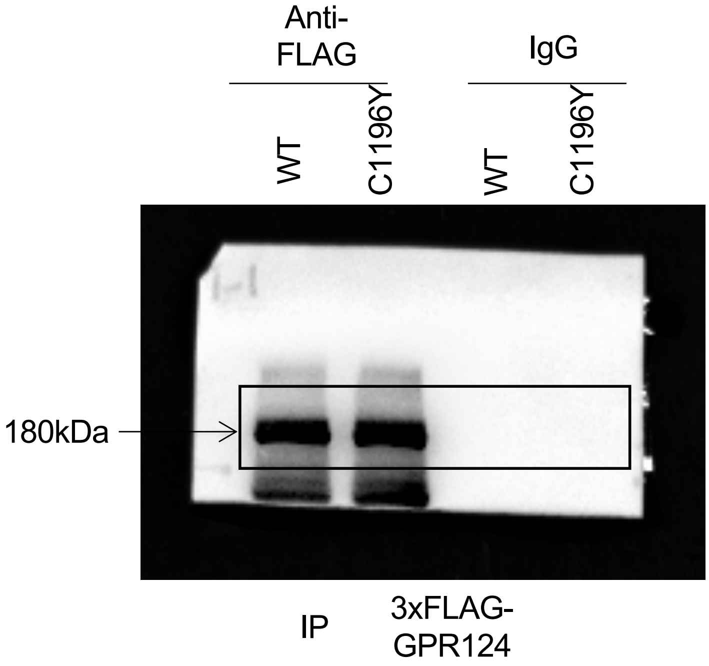

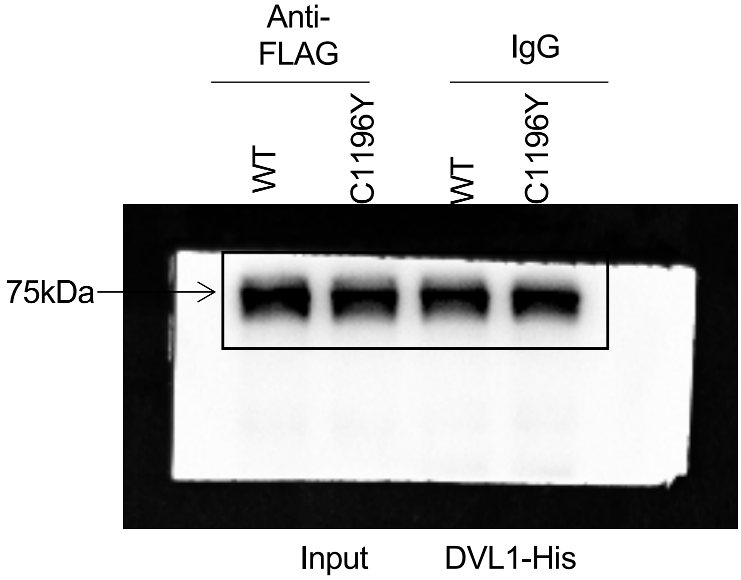

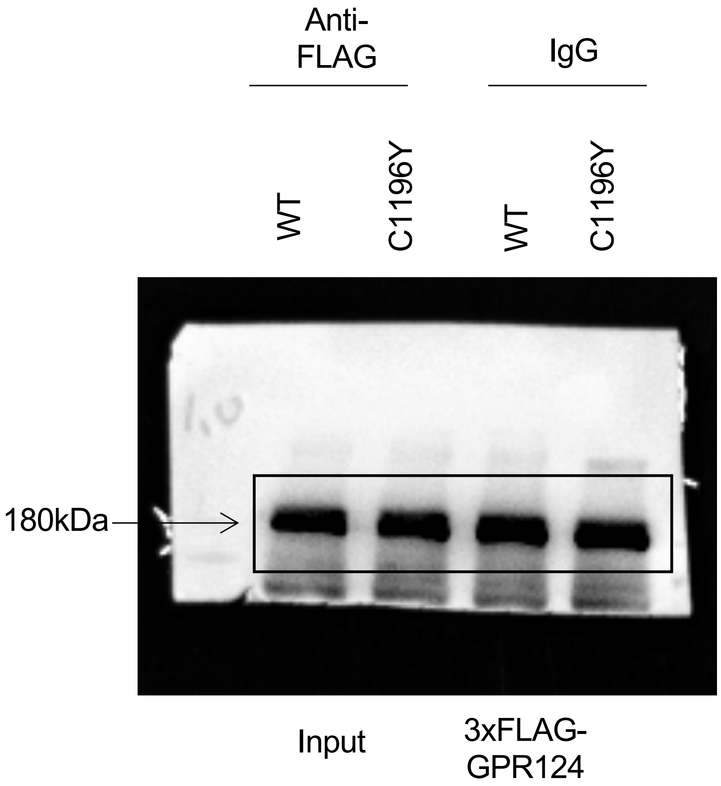

Full uncropped gel/blot for Figure 4E

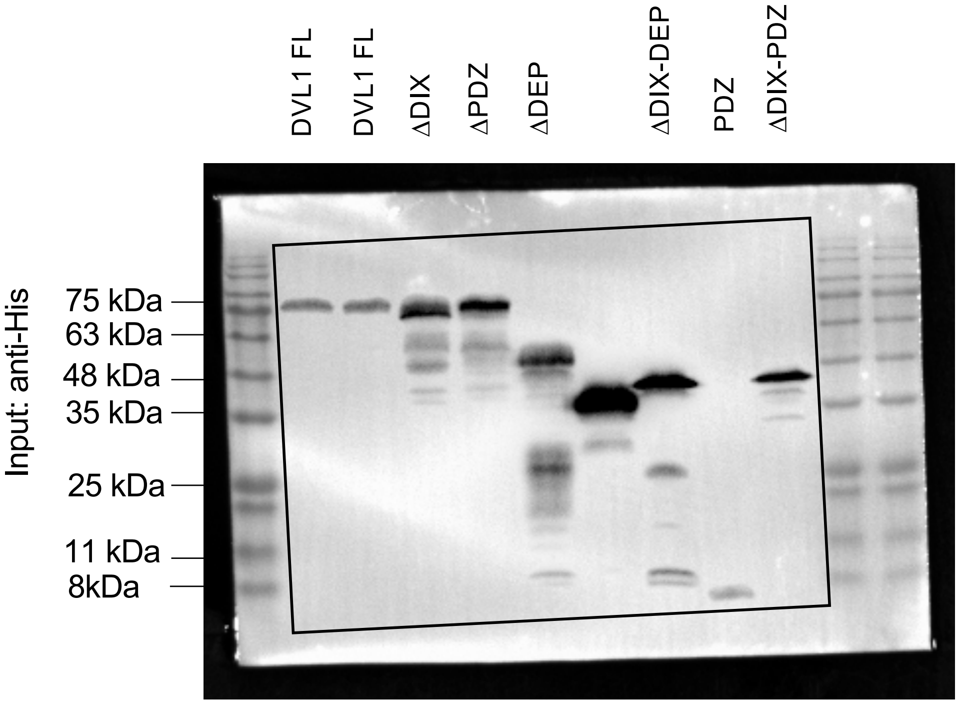

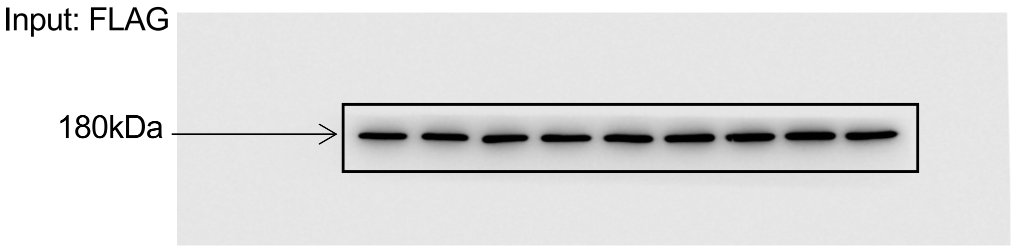

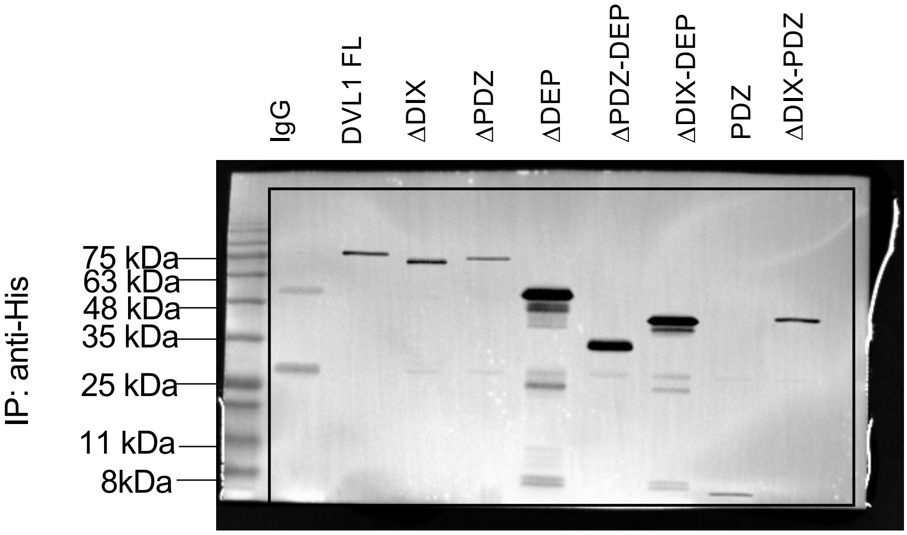

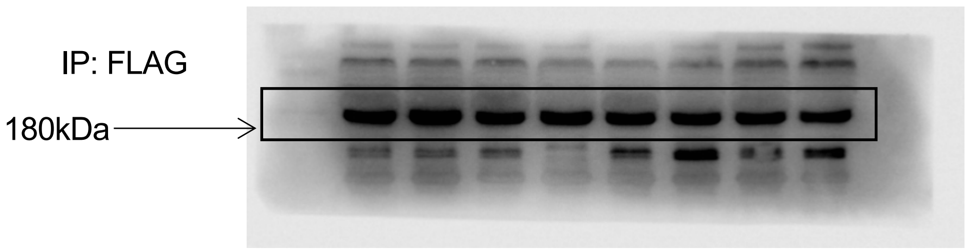

Full uncropped gel/blot for Supplementary Figure 1A

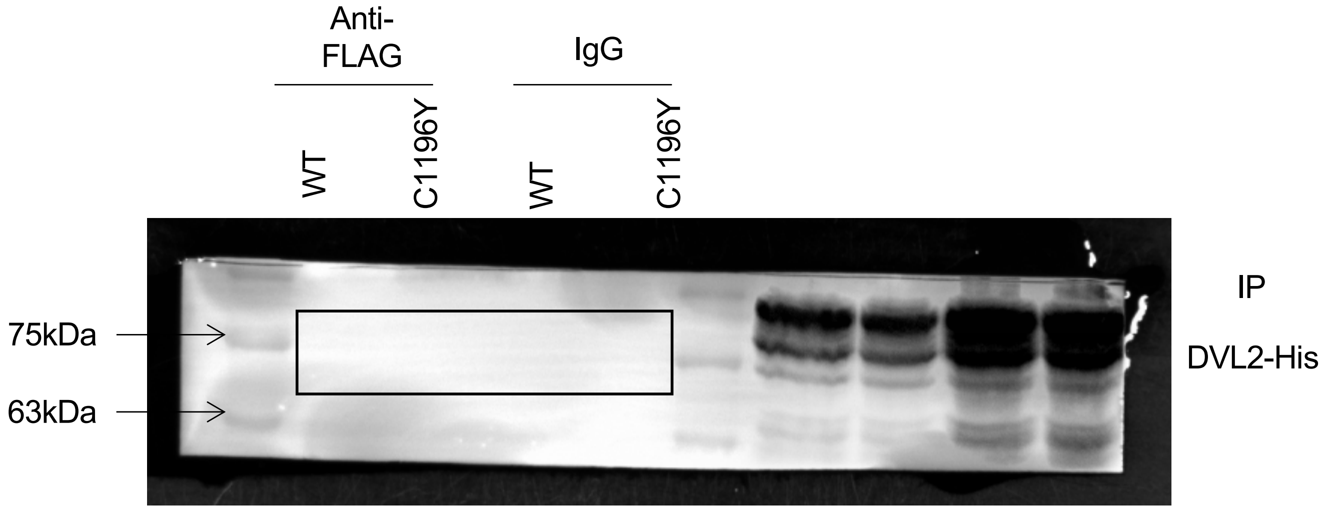

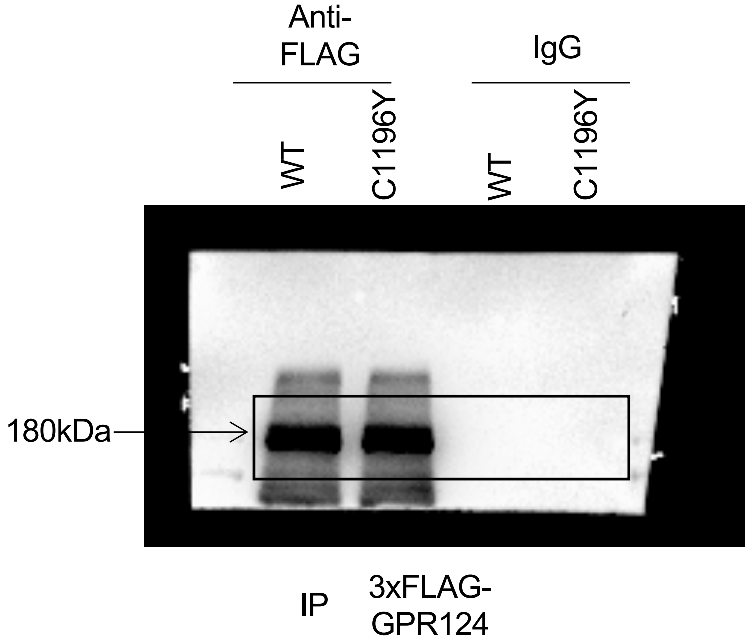

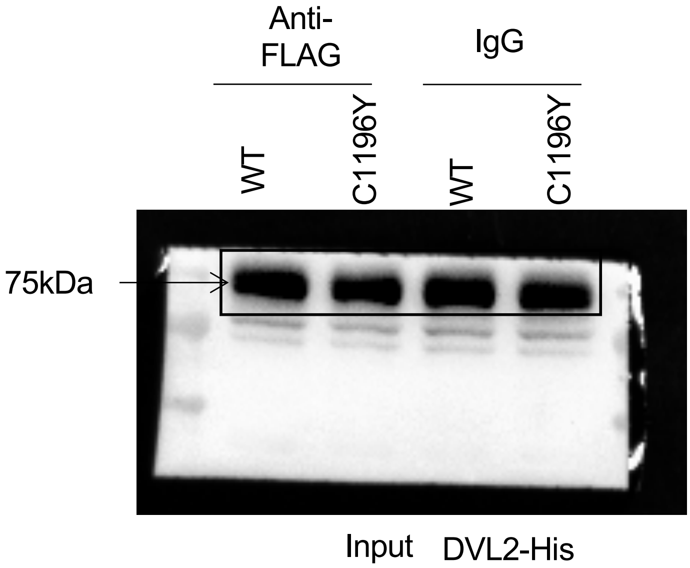

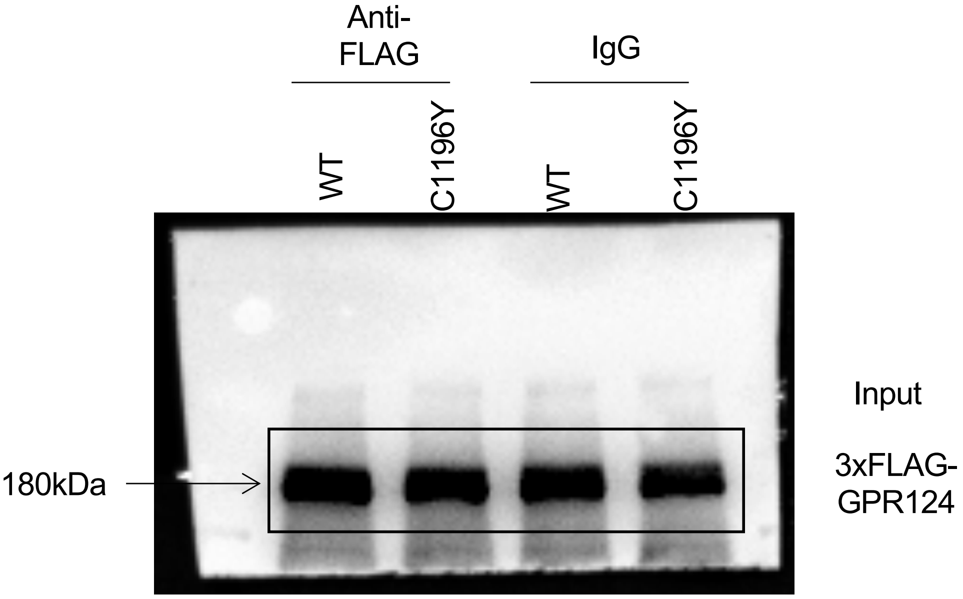

Full uncropped gel/blot for Supplementary Figure 1B

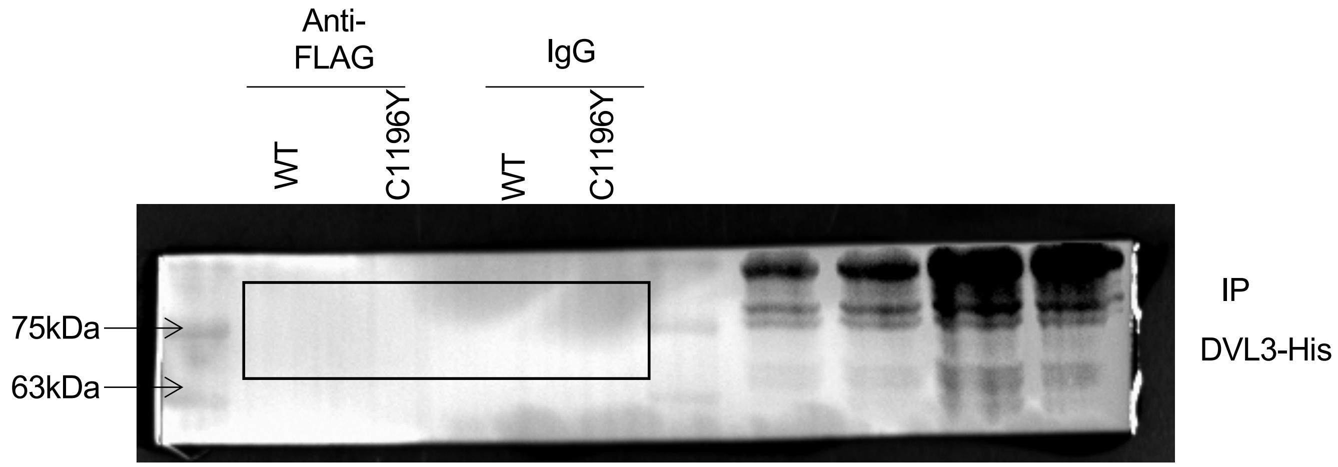

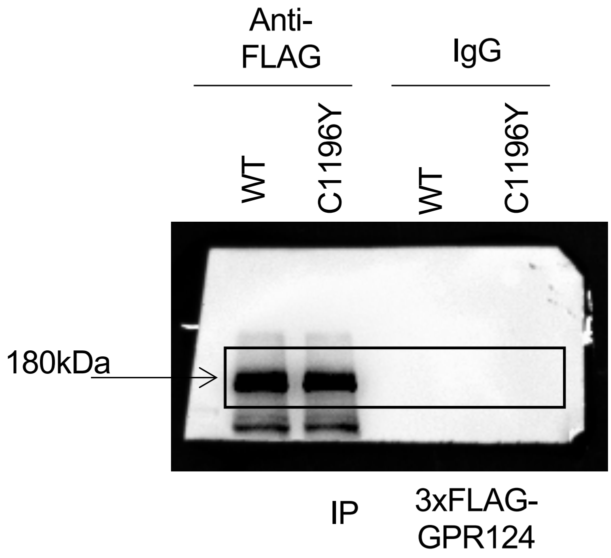

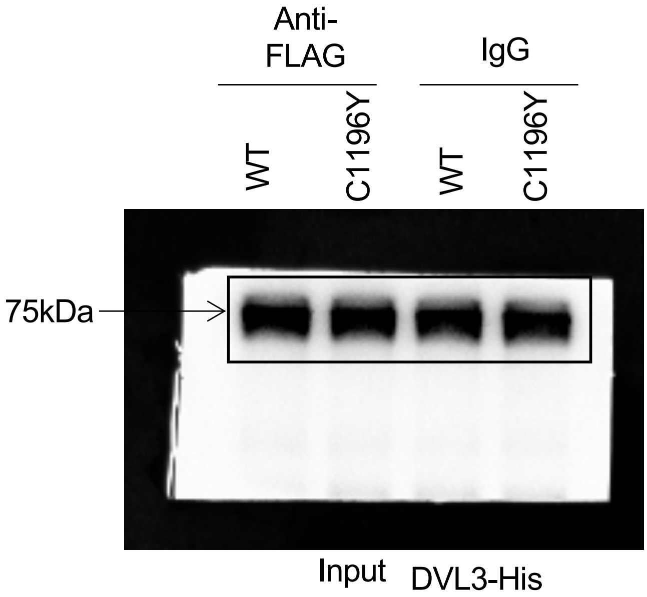

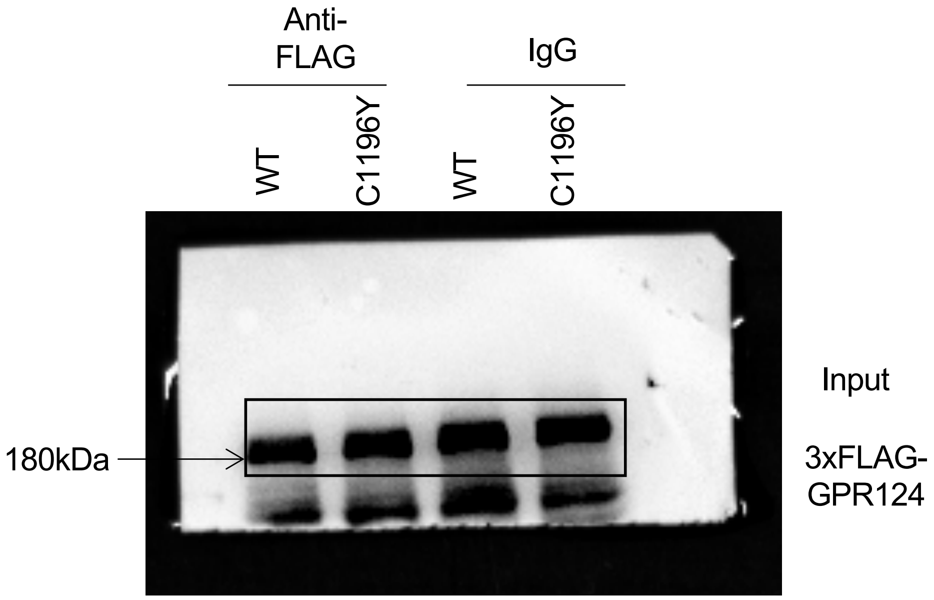

Supplement: Supplementary file 1 — Appendix S1 [file CNS-27-71-s001.pdf]
